# Genomic organization and classification of the bovine WC1 genes and expression by peripheral blood gamma delta T cells

**DOI:** 10.1186/1471-2164-10-191

**Published:** 2009-04-24

**Authors:** Carolyn TA Herzig, Cynthia L Baldwin

**Affiliations:** 1 Department of Veterinary and Animal Sciences, University of Massachusetts, Amherst, MA, 01003, USA; 2 Program in Molecular and Cellular Biology, University of Massachusetts, Amherst, MA, 01003, USA

## Abstract

**Background:**

WC1 co-receptors are group B scavenger receptor cysteine-rich molecules that are found exclusively on γδT cells and are thought to be encoded by a multi-gene family. Previous studies have shown γδT cells that respond to a particular stimulus have unique WC1 molecules expressed. Prior to the onset of the studies described here only one full-length WC1 nucleotide sequence was publicly available, though three WC1 molecules had been distinguished based on monoclonal antibody reactivity. Furthermore, the number of WC1 genes found in the bovine genome and their sequences had not yet been resolved.

**Results:**

By annotating the bovine genome Btau_3.1 assembly, here we show the existence of 13 members in the WC1 gene family and their organization within two loci on chromosome 5 including three distinct exon-intron gene structures one of which coded for a potentially more primitive and smaller WC1 molecule that is similar to the swine WC1 gene. We also provide cDNA evidence as verification for many of the annotated sequences and show transcripts for isoforms derived by alternative splicing.

**Conclusion:**

It is possible that WC1 diversity contributes to functional differences that have been observed between γδT cell populations. The studies described here demonstrate that WC1 molecules are encoded by a large, multi-gene family whose transcripts undergo extensive alternative splicing. Similar to other non-rearranging immunoreceptors, it is likely that the WC1 gene repertoire underwent expansion in order to keep pace with rapidly changing ligands.

## Background

γδT cells play important roles in immune responses by their capacity to produce IFN-γ [1,2], down-modulate immune responses following pathogen clearance [3,4] and develop recall responses to antigen [1,5-7]. Thus it is important to understand the manner by which they are engaged in immune responses. Workshop cluster 1 (WC1) co-receptors are group B scavenger receptor cysteine-rich (SRCR) transmembrane glycoproteins that are unique to γδT cells [8-10] of some species including cattle [11], sheep (T19) [9,12] and swine [13], but have not yet been identified for humans or mice. Since different molecular forms of WC1 are found on functionally distinct subpopulations of bovine γδT cells [2,14], we propose it to be a pattern recognition molecule and/or involved in regulating signaling through the T cell receptor (TCR). Support for this comes from other group B SRCR molecules found on immune system cells including CD5 which is a positive and negative regulator of TCR and B cell receptor signaling [15] and CD6 which functions as an adhesion molecule [16] and for activation [17-19]. Also CD163, predominantly expressed on monocytes and macrophages [20], plays an important role in down-regulating inflammatory responses [21] while DMBT1 on macrophages functions as a pattern recognition molecule (for review see [22]).

Diversity among the immune system-associated SRCR molecules can result from alternative splicing of their individual genes. CD5 and CD6 undergo alternative splicing within the cytoplasmic tail region [23-25] but function of these isoforms is unclear. Both CD163 and DMBT1 undergo extensive alternative splicing of the extracellular and cytoplasmic coding regions, and a CD163 isoform lacking the transmembrane region has been described [22,26,27]. While the predominant short-tailed CD163 variant mediates ligand internalization and degradation, the function of the long-tail CD163 variant is unknown [28] as is the role of DMBT1 splice variants. Alternatively spliced variants have also been reported for swine and sheep WC1 orthologs, splicing occurs within both extracellular and cytoplasmic domains, and isoforms lacking the transmembrane region have been identified [29,30]. Thus while isoform formation appears to be common among SRCR molecules, diversity of immunoreceptors can also result from multiple genes and in this regard WC1 is distinguished from the others.

Although WC1 is unique among group B SRCR molecules because it alone is coded for by a large multi-gene family [23,24,26,31,32], other non-SRCR immunoreceptors are coded for by multi-gene families including the C-type lectin-like Ly49. Ly49 is a major receptor that regulates natural killer (NK) cells and some T cells. It has 15 functional genes in mice [33,34], but only a single copy in humans [35,36] and cattle [37]. Additionally, the killer Ig-like receptor (KIR) multi-gene family found on NK and T cells of primates [38,39] and cattle [37] encodes products that are functionally similar to but structurally distinct from those of the Ly49 family. Both the KIR and Ly49 gene families are believed to have resulted from rapid repeated gene duplication [33,40,41]. It has been speculated that these families of closely related non-rearranging immunoreceptors have evolved to include large numbers of duplicated genes to keep pace with rapidly changing ligands. This appears to be the case for MHC class I molecules, which comprise a large family and are the ligands for Ly49 receptors in mice and rats and for KIR family members in cattle and primates [33,37,42,43]. We hypothesize that in the case of γδT cells WC1 is similarly important in inducing and perpetuating γδT cell responses thus necessitating the expansion of the WC1 repertoire. This is supported by previous studies showing that antigen responsive WC1^+ ^γδT cells express a restricted set of TCR gamma genes regardless of the WC1 molecule found on the cells [44] and that subpopulations of γδT cells that respond to different stimuli express different WC1 molecules [2,14].

While three distinct WC1 molecular forms have been distinguished based on partial sequence and their reactivity with monoclonal antibodies (mAb) [45], the number of WC1 genes found in the bovine genome and their sequences has yet to be resolved. Only one full-length nucleotide sequence is publicly available (i.e. WC1.1, known as the archetypal WC1) [11]. For sheep WC1 (also known as T19), over 50 genes are predicted [46,47] based on Southern blot analysis while for cattle many related genes are predicted based on Southern blots [11] and 13 based on cDNA sequences of the WC1 intracytoplasmic tails [48]. While the number of genes coding for porcine WC1 proteins is unknown, swine have a more abbreviated version of WC1 which has 6 SRCR domains. This suggests swine WC1 genes did not undergo the same internal SRCR domain-duplication that occurred in cattle [29] since previously described bovine WC1 molecules have 11 SRCR domains [11]. By annotating the bovine genome Btau_3.1 assembly, here we demonstrate the existence of 13 members in the WC1 family and show their organization within two loci including a bovine gene coding for the putatively more primitive and smaller swine-like WC1 molecule. We also provide cDNA evidence as verification for many of the annotated sequences as well as for isoforms derived by alternative splicing.

## Results

### Annotation of WC1 genes

**Figure 1:**
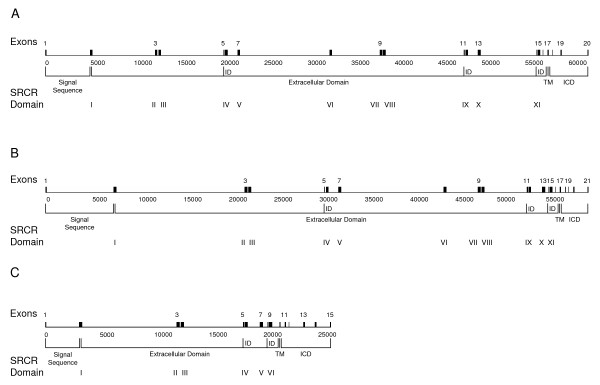
**Schematic representation of WC1 exon-intron structure**. Three structures were identified and were found to contain differing numbers of exons. Representative structures of WC1 genes containing (A) 20 exons (Type I; based on genomic sequence for *WC1-5*, GLEAN_13176) (B) 21 exons (Type II; based on genomic sequence for *WC1-9*, GLEAN_12191) (C) 15 exons (Type III, based on genomic sequence for *WC1-11*, GLEAN_09904 and GLEAN_12182) are shown. Exon numbers and SRCR domain numbers are indicated. Scale is shown in base pair increments beneath the schematic. Abbreviations are as follows: ID, inter-domain sequence; TM, transmembrane region; ICD, intracytoplasmic domain.

Here we set out to annotate the bovine genome to confirm the existence of the large number of WC1 genes (10–13 genes) transcript analyses supported for cattle [11,48]. While diversity in cattle WC1 molecules could result from alternative gene splicing as occurs for another T cell expressed-SRCR gene CD6 [23], Southern blots indicated up to 50 WC1 genes in the closely related ruminant species of sheep [46,47]. It is known that in ruminants WC1 SRCR domains are each encoded by a single exon and that at least archetypal WC1.1 consists of 11 SRCR domains, the first of which is a unique Domain 1 followed by a region of 5 SRCR domains that is repeated once [11]. Archetypal WC1.1 also has three inter-domain regions and a transmembrane region that are encoded by single exons and a cytoplasmic tail coded by either 4 or 5 exons [2]. Using this paradigm as a starting point, we identified three types of WC1 genes based on unique exon-intron structures (Figure 1). All three types contained exons coding for highly-related repeating SRCR domains, inter-domain sequences, transmembrane domains and intracytoplasmic tail domains. The distinguishing features between the types represented by the schematic in Figure 1A (Type I) and Figure 1B (Type II) is that Type I genes contained one less exon than Type II genes for a total of 20 or 21 exons, respectively. This additional exon in Type II was found within the intracytoplasmic coding sequence and accounts for the "long-tail" WC1 molecular form [2] which can have 15 to more amino acids in the intracytoplasmic tail region than are found in Type I genes. Both of the types shown in Figure 1A and Figure 1B demonstrates that WC1 genes encompass a coding region of approximately 60 kb. This is in contrast with the third type of WC1 genes identified here (Type III, Figure 1C), which contained 15 exons total and encompassed a coding region of approximately 25 kb. Type III genes code for a WC1 molecule structurally similar to the described swine WC1 with 6 SRCR domains [29]. Unlike Type I and Type II genes, whose intracytoplasmic domains are encoded by four or five exons, respectively, Type III genes appear to have a much longer intracytoplasmic domain which is encoded by six exons resulting in approximately 80 additional acids relative to Type I.

**Figure 2:**
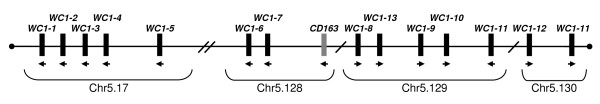
**Schematic representation of WC1 loci organization**. 13 WC1 genes were found within two loci on chromosome 5 with *CD163*, another group B SRCR molecule, found within one of the WC1 loci. WC1 gene designations, orientations and Bovine Genome Scaffold identifications are as indicated. Diagram is not shown to scale. *WC1-11 *was identified in two gene prediction models; however, evidence suggests that there is only a single *WC1-11 *gene.

**Figure 3:**
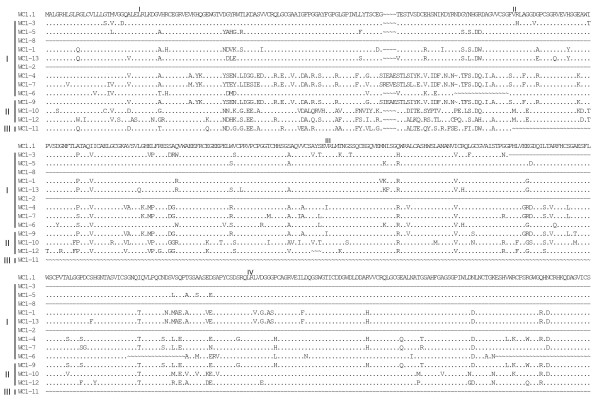
**Alignment of WC1 deduced amino acid sequences identified in the genome (part 1)**. Full-length deduced amino acid sequences of the annotated WC1 genes were aligned using ClustalW2 and the default parameters and were refined by hand. The archetypal WC1 sequence (WC1.1, GenBank accession number X63723) was included in the analysis for comparison. *WC1-2*, *WC1-3*, *WC1-6*, *WC1-8 *and *WC1-12 *sequences are partial due to gaps in the genome sequence. Gene types (I, II or III), as determined based on exon-intron structure are indicated to the left of the sequences. Identities are indicated by dots (.), gaps resulting from the alignment are indicated by tildes (~), gaps resulting from lack of genomic sequence (when the gaps were found adjacent and not within a coding region) are indicated by dashes (-). SRCR domains are indicated in roman numerals and the transmembrane region is shown underlined for the archetypal WC1.1 sequence.

**Figure 4:**
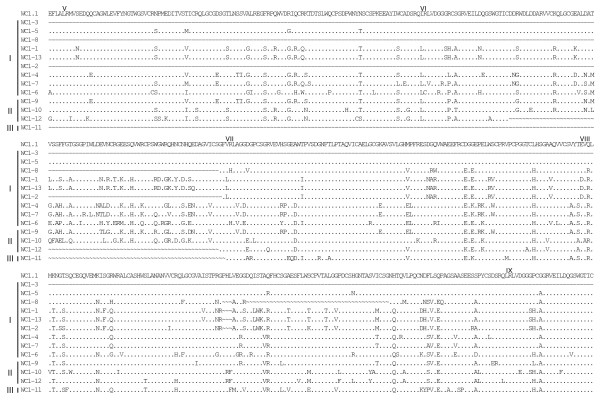
**Alignment of WC1 deduced amino acid sequences identified in the genome (part 2)**. Full-length deduced amino acid sequences of the annotated WC1 genes were aligned using ClustalW2 and the default parameters and were refined by hand. The archetypal WC1 sequence (WC1.1, GenBank accession number X63723) was included in the analysis for comparison. *WC1-2*, *WC1-3*, *WC1-6*, *WC1-8 *and *WC1-12 *sequences are partial due to gaps in the genome sequence. Gene types (I, II or III), as determined based on exon-intron structure are indicated to the left of the sequences. Identities are indicated by dots (.), gaps resulting from the alignment are indicated by tildes (~), gaps resulting from lack of genomic sequence (when the gaps were found adjacent and not within a coding region) are indicated by dashes (-). SRCR domains are indicated in roman numerals and the transmembrane region is shown underlined for the archetypal WC1.1 sequence.

**Figure 5:**
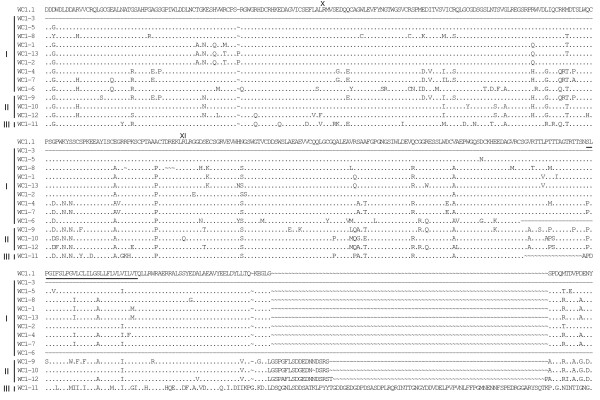
**Alignment of WC1 deduced amino acid sequences identified in the genome (part 3)**. Full-length deduced amino acid sequences of the annotated WC1 genes were aligned using ClustalW2 and the default parameters and were refined by hand. The archetypal WC1 sequence (WC1.1, GenBank accession number X63723) was included in the analysis for comparison. *WC1-2*, *WC1-3*, *WC1-6*, *WC1-8 *and *WC1-12 *sequences are partial due to gaps in the genome sequence. Gene types (I, II or III), as determined based on exon-intron structure are indicated to the left of the sequences. Identities are indicated by dots (.), gaps resulting from the alignment are indicated by tildes (~), gaps resulting from lack of genomic sequence (when the gaps were found adjacent and not within a coding region) are indicated by dashes (-). SRCR domains are indicated in roman numerals and the transmembrane region is shown underlined for the archetypal WC1.1 sequence.

**Figure 6:**
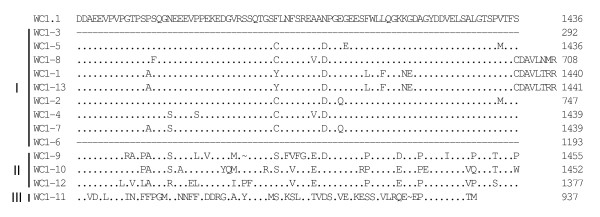
**Alignment of WC1 deduced amino acid sequences identified in the genome (part 4)**. Full-length deduced amino acid sequences of the annotated WC1 genes were aligned using ClustalW2 and the default parameters and were refined by hand. The archetypal WC1 sequence (WC1.1, GenBank accession number X63723) was included in the analysis for comparison. *WC1-2*, *WC1-3*, *WC1-6*, *WC1-8 *and *WC1-12 *sequences are partial due to gaps in the genome sequence. Gene types (I, II or III), as determined based on exon-intron structure are indicated to the left of the sequences. Identities are indicated by dots (.), gaps resulting from the alignment are indicated by tildes (~), gaps resulting from lack of genomic sequence (when the gaps were found adjacent and not within a coding region) are indicated by dashes (-). SRCR domains are indicated in roman numerals and the transmembrane region is shown underlined for the archetypal WC1.1 sequence.

Using these three gene structures as a basis for annotation of GLEAN gene-prediction models from the Btau_3.1 assembly, the organization and orientation of 13 WC1 genes on chromosome 5 was determined (Figure 2). WC1 genes were identified on four scaffolds including Chr5.17, Chr5.128, Chr5.129 and Chr5.130 and chromosomal locations are reported in Table 1. Annotated genomic WC1 sequences were classified and named generally based on chromosomal location. The following GLEAN gene models were identified by BLAST search using the archetypal WC1 sequence and were manually annotated as WC1 genes: GLEAN_13183 (*WC1-1*), GLEAN_13182 (*WC1-2*), GLEAN_13181 (*WC1-3*), GLEAN_13179 (*WC1-4*), GLEAN_13176 (*WC1-5*), GLEAN_00457/GLEAN_00458 (*WC1-6*), GLEAN_00456 (*WC1-7*), GLEAN_12186 (*WC1-8*), GLEAN_12191 (*WC1-9*), GLEAN_12192 (*WC1-10*), GLEAN_12182 (*WC1-11*), GLEAN_09904 (*WC1-11*), GLEAN_09902 (*WC1-12*), GLEAN_12187 (*WC1-13*). The presence of two GLEAN gene models for *WC1-11 *was noted; however, the genomic sequence found within the introns and flanking the genes for both models was identical which is indicative of an assembly anomaly. Because the two models were found in opposite orientations of one another it is possible that scaffold Chr5.130 itself was assembled in the wrong orientation. In some cases gaps in the genomic sequence made it impossible to annotate a complete gene and therefore some of the sequences are incomplete (see Figures 3, 4, 5 and 6). Specifically, sequences were incomplete due to gaps adjacent to the coding region (*WC1-2*, *WC1-3 *and *WC1-8*), gaps within the coding region (*WC1-12*) or because the gene was situated at the end of a scaffold (*WC1-6*). Also, sequences coding for additional SRCR domains were found both outside and within the coding regions of putative WC1 genes and were predicted to be either pseudogenes (not shown) or part of intronic sequence based on the presence of premature stop codons or frameshifts that resulted in premature stop codons. Nevertheless, the data presented here support the existence of a large family of genes, with three distinct structures, coding for individual WC1 molecules in ruminants.

**Table 1:** Chromosomal location of WC1 genes

**Gene designation**	**GLEAN number**	**Scaffold**	**Start**	**End**	**Orientation**
*WC1-1*	GLEAN_13183	Chr5.17	95127	43521	-
*WC1-2*	GLEAN_13182	Chr5.17	139700^a^	117360	-
*WC1-3*	GLEAN_13181	Chr5.17	219521	201515^a^	-
*WC1-4*	GLEAN_13179	Chr5.17	329920	268868	-
*WC1-5*	GLEAN_13176	Chr5.17	563956	501522	-
*WC1-6*	GLEAN_00457/GLEAN_00458	Chr5.128	58221	1425^b^	-
*WC1-7*	GLEAN_00456	Chr5.128	129730	71150	-
*WC1-8*	GLEAN_12186	Chr5.129	260959^a^	285020	+
*WC1-9*	GLEAN_12191	Chr5.129	425569	485506	+
*WC1-10*	GLEAN_12192	Chr5.129	496564	546669	+
*WC1-11*	GLEAN_12182	Chr5.129	634583	608420	-
*WC1-11*	GLEAN_09904	Chr5.130	332841	359001	+
*WC1-12*	GLEAN_09902	Chr5.130	18079	76442	+
*WC1-13*	GLEAN_12187	Chr5.129	298669	350030	+
*CD163*	GLEAN_00453	Chr5.128	391036	359955	-

^a ^Sequence incomplete due to gap adjacent to coding region.

^b^Sequence incomplete due to scaffold end.

Deduced amino acid sequences of the 13 annotated WC1 genes described here were aligned and shown compared with the archetypal WC1 sequence (Figures 3, 4, 5 and 6). Genes are grouped according to their distinct gene structure (i.e. Types I – III) and it is evident that genes of a particular type have sequence similarity. However, despite *WC1-4 *and *WC1-9 *having different types of gene structures their extracellular regions are very similar although differences within their intracytoplasmic tail regions occur as would be expected since *WC1-9 *has an extra exon. When WC1 gene distribution on chromosome 5 was evaluated it was found that their relatedness based on type did not correlate entirely with their genomic location (refer to Figure 2).

### cDNA evidence for WC1 distinct gene structures

mRNA derived from bovine PBMC was analyzed to confirm the transcription of the multiple genes annotated above. To verify that cDNA sequences (designated with a prefix of 'CH') obtained were indeed representative of multiple genes, instead of genetic variation among animals, all reactions were performed using mRNA from a single animal. Also, because WC1^+ ^γδT cells with different WC1 molecular forms respond to different activation stimuli [2,14], reactions were performed with pooled cDNA from ConA-activated, leptospira-activated and ex vivo PBMC in order to maximize the number of WC1 transcripts amplified.

Analysis of PCR products showed that primer combinations (see Table 2 and Methods) used to amplify full-length WC1 yielded two bands of approximately 4.4 kb and 2.7 kb for primer pairs WC1atg-for/WC1group1,2-rev and WC1atg-for/WC1group4-rev and approximately 2.9 kb and 2.2 kb for primer pair WC1atg-for/WC1group3-rev. Sequencing revealed that the larger bands of approximately 4.4 kb were most frequently full-length WC1 of Type I and II while the 2.9 kb was most frequently full-length Type III. However, on occasion some transcripts in these bands were found to be alternatively spliced. The smaller sized bands of approximately 2.7 kb and 2.2 kb contained transcripts that always represented alternative splicing and are described further below. Sequence alignments were generated based on the deduced amino acid sequences from representative cDNA clones and annotated WC1 genes and percent identities were calculated based on those alignments (Table 3). Because the sequences varied in length due to either incomplete genomic sequence availability or alternative splice variation, sequences were aligned in a pairwise manner and were truncated so that comparisons were made only between regions that were common to both sequences. Archetypal WC1 sequence (GenBank accession number X63723) was compared to both the annotated WC1 gene sequences as well as the cDNA sequences generated as part of this study. This analysis revealed that while primer pairs WC1atg-for/WC1group1,2-rev and WC1atg-for/WC1group4-rev amplified the same transcripts, primer pair WC1atg-for/WC1group3-rev specifically amplified cDNA whose sequence represented Type III genes and thus was most similar to swine WC1 (GenBank accession number CAA67709). This also demonstrated that cDNA evidence was available for many of the predicted genes including ones representing each of the three types including *WC1-1*, *WC1-3*, *WC1-4 *and *WC1-13 *as Type I, *WC1-9 *as Type II and *WC1-11 *as Type III.

**Table 2:** Primers used for WC1 transcript amplification and for cDNA clone sequencing reactions

**Primer name**	**Sequence (5'-3')**
Primers used for gene amplification:	
WC1atg-for	ATGGCTCTGGGCAGACACCTCTC
WC1full-forA	GATGGTTACAGGTGGACATTGAAGG
WC1group1,2-rev	TCAYGAGAAAGTCAYTGKGGATG
WC1group3-rev	CTACATGGTGCTAAGCTCCACATC
WC1group4-rev	TCATCTCCTAGTTAATACAGCATC
SRCR1-rev	TGCACAGATGACCTGGGCAGTGG
Primers used for sequencing reactions:	
WC1seq1-for	CTCAACCTACAGGCTCTG
WC1seq2-for	CAGGTGGAGATGAACATTTC
WC1seq3-for	CACGACTGCAGACACAAGC
WC1seq4-for	GAAGACATCACTGTGTCCGTG
WC1seq5-for	GAGAAGCCCTCAATGCCAC
WC1seq6-for	CATGGAAGACATCACTGTG
WC1seq7-for	GCTACACAGATGGAGAGCAG
WC1seq8-for	GGCTCATGACAAACGGCTCCTCTCAG
WC1seq1-rev	GAGCTTCTCTCTGTCTGTGCAG
WC1seq2-rev	GCAGCACAGTCCCACAG
WC1seq3-rev	GCCCTTCCCCAAAGTGAGCTG
WC1seq4-rev	CTGGTCAAGGATCTCCACTCTC
WC1seq5-rev	GATCCTGCCCCAAAGTGAGC
WC1seq6-rev	GTGTGAAGTTTCCATCAGAC
WC1seq7-rev	GCAGTAGGGAGAACTCTC

**Table 3:** cDNA evidence for transcription of all three WC1 distinct gene structure types^a^

		**cDNA clone**^b^							
		
**Gene Designation**		**WC1.1**	**CH534**	**CH501**	**CH496**	**CH505**	**CH504**	**CH486**	**CH525**
**Type I**	**WC1.1**	X	99.0	92.3	85.3	82.0	88.5	67.4	95.3
	***WC1-3***	92.1	**91.8**	87.0	74.0	73.0	86.0	77.7	86.6
	***WC1-5***	97.4	97.1	92.1	85.8	82.6	88.7	67.5	95.6
	***WC1-8***	90.6	90.1	90.5	87.9	80.5	89.2	66.1	91.1
	***WC1-1***	89.0	88.3	**98.0**	83.8	80.6	93.5	67.9	89.7
	***WC1-13***	88.5	87.8	96.2	83.6	80.4	**93.8**	67.6	88.9
	***WC1-2***	91.2	91.1	94.8	87.1	81.0	94.6	66.3	97.0
	***WC1-4***	85.7	85.1	84.8	**98.7**	93.0	84.0	67.4	84.4
	***WC1-7***	85.8	85.1	84.8	94.9	90.1	84.2	67.3	84.3
	***WC1-6***	83.8	85.6	88.1	82.2	82.2	84.6	79.2	86.9
									
**Type II**	***WC1-9***	82.2	81.6	78.6	92.9	**98.5**	80.4	67.2	79.5
	***WC1-10***	83.1	82.6	80.4	81.7	82.7	81.3	69.5	80.5
	***WC1-12***	83.6	83.0	81.1	81.4	83.2	81.3	68.9	80.4
									
**Type III**	***WC1-11***	67.7	67.5	67.7	67.7	67.7	66.9	**99.1**	67.1

^a ^Percent identity is shown for the deduced amino acid sequences of WC1 genes and cDNA clones.

^b ^Bold values indicate cDNA clones classified as WC1 genes.

### Domain 1 is the most variable SRCR domain

When we compared individual WC1 SRCR domains using cDNA and annotated genomic sequences by aligning deduced amino acid sequences we found the greatest variability among Domain 1's, with percent identities as low as 50.4% (Table 4). There was less variability among the other domains with Domain 9's sharing the most identity ranging from 88.2% to 99.0% (Table 4). Classification of WC1 cDNA clones described here, based on exon-intron structure and on sequence identity to annotated WC1 sequences, is reported in Table 5 based on the following reasoning. In many cases sequence identity of ≥ 98%, based on comparison of full-length sequence as described in Table 3, was sufficient to classify cDNA sequences: CH501 as *WC1-1*, CH496 as *WC1-4*, CH505 as *WC1-9 *and CH486 as *WC1-11*. In every case, except for CH486, additional WC1 genes shared > 90% identity with classified cDNA sequences.

**Table 4:** Deduced amino acid sequence percent identity within individual, non-redundant WC1 domains

**Domain**	**Number of sequences compared**^a^	**Highest percent identity**	**Lowest percent identity**
1	21	99.0	50.4
2	16	99.0	74.2
3	20	99.0	81.5
4	13	99.0	85.2
5	18	99.0	76.1
6	14	99.0	69.6
7	13	99.0	79.4
8	12	99.0	73.7
9	14	99.0	88.2
10	17	99.1	79.8
11	21	99.0	80.3

^a ^Sequences used for analysis included WC1 genomic sequences and cDNA sequences. For each domain, all sequences were compared and redundant sequences were removed from the analysis.

**Table 5:** WC1 cDNA sequences and classification based on annotated genomic sequences

**cDNA clone name**	**GenBank accession number**	**Exon-intron structure type**	**WC1 classification**
CH501	FJ031186	Type I	*WC1-1*
CH504	FJ031187	Type I	*WC1-13*
CH503	FJ031188	Type I	*WC1-13*
CH481	FJ031189	Type I	*WC1-13*
CH499	FJ031190	Type I	*WC1-13*
CH534	FJ031191	Type I	*WC1-3*
CH521	FJ031192	Type I	*WC1-3*
CH533	FJ031193	Type I	*WC1-3*
CH520	FJ031194	Type I	*WC1-3*
CH527	FJ031195	Type I	*WC1-3*
CH528	FJ031196	Type I	*WC1-3*
CH453	FJ031197	Type I	*WC1-3*
CH455	FJ031198	Type I	*WC1-3*
CH461	FJ031199	Type I	*WC1-3*
CH465	FJ031200	Type I	*WC1-3*
CH469	FJ031201	Type I	*WC1-3*
CH496	FJ031202	Type I	*WC1-4*
CH507	FJ031203	Type I	*WC1-4*
CH506	FJ031204	Type I	*WC1-4*
CH497	FJ031205	Type I	*WC1-4*
CH482	FJ031206	Type I	*WC1-4*
CH526	FJ031207	Type I	*WC1-4*
CH505	FJ031208	Type II	*WC1-9*
CH486	FJ031209	Type III	*WC1-11*
CH487	FJ031210	Type III	*WC1-11*
CH485	FJ031211	Type III	*WC1-11*
CH492	FJ031212	Type III	*WC1-11*
CH489	FJ031213	Type III	*WC1-11*
CH490	FJ031214	Type III	*WC1-11*
CH491	FJ031215	Type III	*WC1-11*
CH525	FJ031216	Type I	*WC1-nd1*^a^
CH523	FJ031217	Type I	*WC1-3*
CH529	FJ031218	Type I	*WC1-13*
CH522	FJ031219	Type I	*WC1-3*
CH535	FJ031220	Type I	*WC1-13*

^a ^nd (not designated) denotes a cDNA sequence for which a genomic equivalent gene has not been identified.

**Figure 7:**
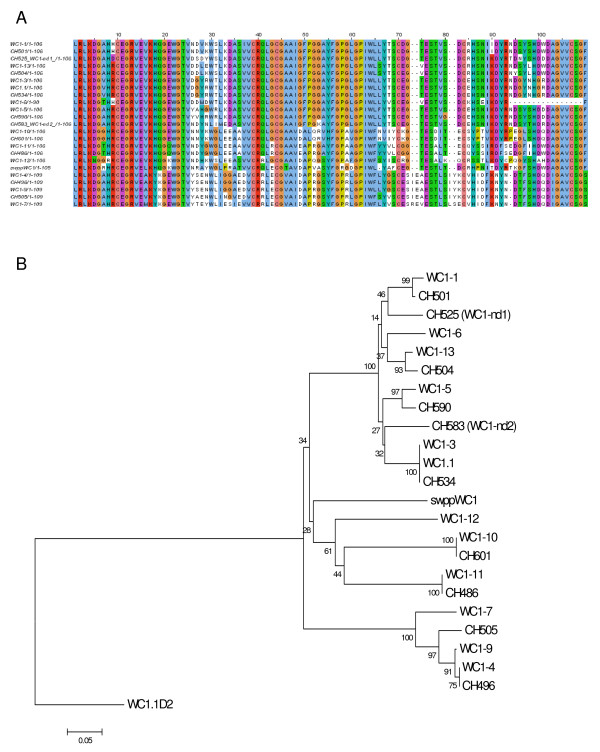
**WC1 Domain 1 sequence**. (A) WC1 Domain 1 deduced amino acid sequences were aligned with ClustalW2 using the default parameters and visualized with JalView. Analysis includes all non-redundant genomic sequences and all non-redundant cDNA sequences. The archetypal WC1 sequence (WC1.1, GenBank accession number X63723) and swine WC1 sequence (swppWC1, GenBank accession number CAA67710) were included in the analysis for comparison. *WC1-6 *sequence is partial due to poor genomic sequence integrity. (B) Phylogenetic tree generated using WC1 Domain 1 deduced amino acid sequences and the Neighbor-Joining method [67]. Archetypal WC1 (WC1.1) and swine WC1 (swppWC1) Domain 1 sequences (accession numbers above) were included for comparison and archetypal WC1 Domain 2 (WC1.1D2) sequence was included to root the tree. The optimal tree with the sum of branch length = 2.02452921 is shown with bootstrap values (based on 1000 replicates) reported next to the branches. Positions containing alignment gaps were eliminated only in pairwise sequence comparisons for a total of 112 positions in the final dataset.

In other cases classification required refinement. That is, because Domain 1's were found to be the most variable domain, cDNA and genomic sequences coding for Domain 1's were aligned (Figure 7A) and used to generate a phylogram (Figure 7B). Only non-redundant sequences were included in the analysis and included those described here as Type II (*WC1-9, WC1 -10, WC1-12 *and CH505), Type III (*WC1-11 *and CH486) and Type I (remaining sequences for which type could be determined). These were useful in classifying cDNA sequences with regard to the annotated sequences. While we found when comparing full-length sequences that CH534 was 97.1% identical to *WC1-5*, its Domain 1 sequence is most identical to *WC1-3 *and was therefore classified as such. Conversely, CH504 was 94.6% identical to *WC1-2 *but was classified here as *WC1-13 *(which was 93.8% identical) because no *WC1-2 *Domain 1 sequence is currently available for comparison. Likewise, CH525 was found to be 97.0% identical to *WC1-2 *but was not classified as such due to lack of Domain 1 sequence and is instead designated as *WC1-nd1*. CH525 was the only complete cDNA clone for which no related genomic WC1 was identified out of a total of 35 sequences evaluated. Therefore our findings indicate that differing WC1 molecular forms are based on germline sequences and are not a result of domain-exon rearrangements among individual WC1 genes as occurs for T cell receptor and immunoglobulin genes. The sequence for *WC1-nd1 *is likely to occur in the assembly gaps and suggests at least one additional WC1 gene.

Domain 1 comparisons also provided evidence for transcription of two more genes. CH590 and CH601 Domain 1 sequences (Wang F, Herzig CTA, Baldwin CL, Telfer JC: Response of bovine γδT cells to Leptospira requires WC1 expression. Genes and Immunity, submitted) corresponded to those of the genes designated *WC1-5 *and *WC1-10*, respectively, for which corresponding full-length transcripts were not found above. Since the relationship between other cDNA and genomic sequences seen in Table 3 were largely maintained when just Domain 1 sequences were compared here (Figure 7), this is reasonable evidence for transcription of *WC1-5 *and *WC1-10*. Finally, one Domain 1 cDNA sequence, CH583, lacked genomic corresponding sequence and thus was named *WC1-nd2 *and suggests a fifteenth WC1 gene.

**Figure 8:**
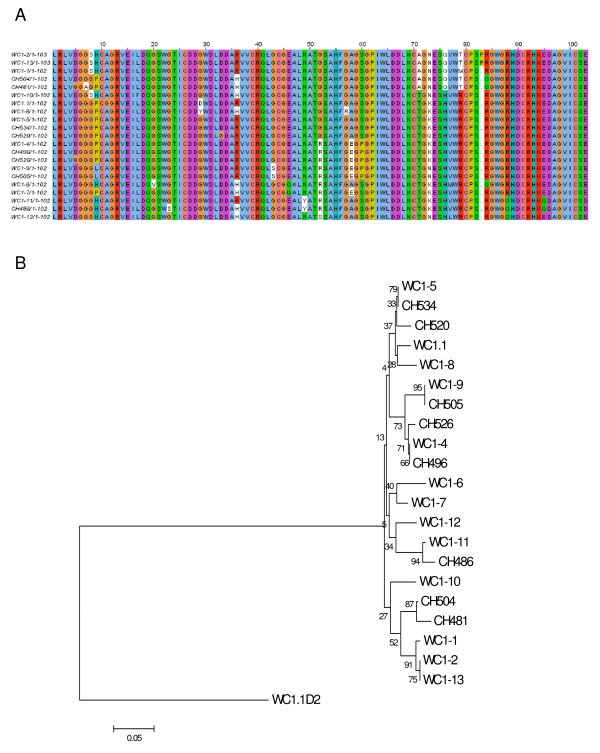
**WC1 Domain 9 sequence**. (A) WC1 Domain 9 deduced amino acid sequences were aligned with ClustalW2 using the default parameters and visualized with JalView. Analysis includes all non-redundant genomic sequences and all non-redundant cDNA sequences. The archetypal WC1 sequence (WC1.1, GenBank accession number X63723) was included in the analysis for comparison. (B) Phylogenetic tree generated using WC1 Domain 9 deduced amino acid sequences and the Neighbor-Joining method [67]. Archetypal WC1 Domain 9 (WC1.1) sequence (accession number above) was included for comparison and archetypal WC1 Domain 2 (WC1.1D2) sequence was included to root the tree. The optimal tree with the sum of branch length = 1.02259498 is shown with bootstrap values (based on 1000 replicates) reported next to the branches. Positions containing alignment gaps were eliminated only in pairwise sequence comparisons for a total of 106 positions in the final dataset.

The phylogram generated based on Domain 1 sequences (Figure 7B) demonstrated that although the genes differ in their exon-intron structures, there was not a similarly clear distinction between the genes when just their Domain 1's were evaluated. However, these data do confirm findings shown in Table 4 indicating that Domain 1 is the most variable domain. This is most notable when seen in contrast with a multiple sequence alignment of deduced amino acid Domain 9 sequences (Figure 8A) and its subsequent phylogram (Figure 8B). Domain 9 was found to be the least variable of the 11 WC1 SRCR domains and this is reflected in the phylogenetic analysis. It is possible that, if Domain 1 is indeed the ligand binding region, selective pressures that drive Domain 1 variability would not necessarily apply to the other SRCR domains.

### Intracytoplasmic region analysis and classification

It could be reasoned that the most divergent of the 11 WC1 SRCR domains, i.e. Domain 1 as shown above, would be the ligand binding region of the WC1 co-receptor and thus that the WC1 Domain 1 sequence associated with a particular γδT cell could influence its response to various stimuli. However, it is also possible that variation in the intracytoplasmic tail regions of WC1 could convey signaling differences which would influence the outcome of receptor ligation. This is supported by previous observations regarding the tail function [49]. Therefore, comparisons of WC1 intracytoplasmic region cDNA sequences with genomic intracytoplasmic sequences were done.

**Figure 9:**
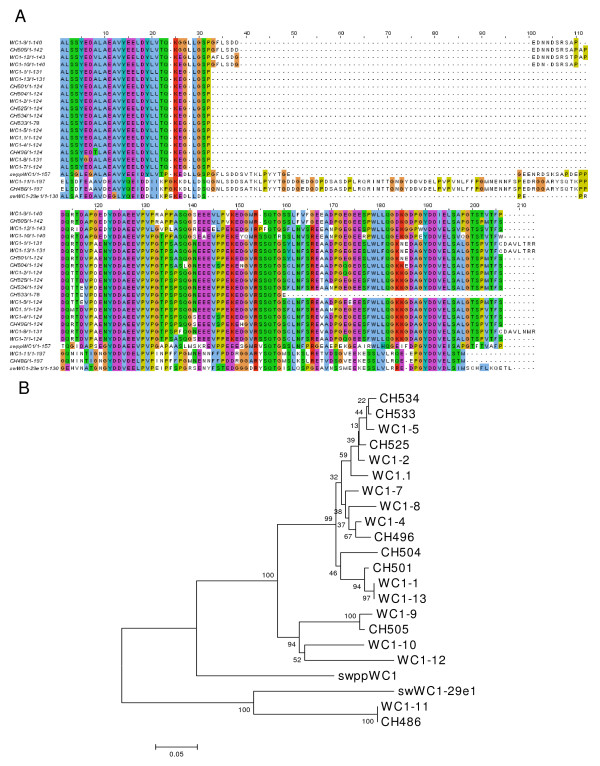
**WC1 intracytoplasmic region sequence**. (A) WC1 intracytoplasmic region deduced amino acid sequences were aligned with ClustalW2 using the default parameters and visualized with JalView. Analysis includes all non-redundant genomic sequences and all non-redundant cDNA sequences and sequences were truncated to begin at the second intracytoplasmic tail encoding exon. The archetypal WC1 (WC1.1, GenBank accession number X63723) and swine WC1 (swppWC1, GenBank accession number CAA67710; swWC1-29e1, GenBank accession number CAA67709) sequences were included in the analysis for comparison. (B) Phylogenetic tree generated using WC1 intracytoplasmic tail deduced amino acid sequences and the Neighbor-Joining method [67]. Archetypal WC1 (WC1.1) and swine WC1 (swppWC1 and swWC1-29e1) intracytoplasmic sequences (accession numbers above) were included for comparison. The optimal tree with the sum of branch length = 1.48497595 is shown with bootstrap values (based on 1000 replicates) reported next to the branches. Positions containing alignment gaps were eliminated only in pairwise sequence comparisons for a total of 213 positions in the final dataset.

The alignment (Figure 9A) and resulting phylogram (Figure 9B) confirmed that tail sequences clustered according to their exon-intron structure (described in Figure 1). That is, *WC1-9*, *WC1-10*, *WC1-12 *and CH505 clustered and are representative of the Type II WC1 molecular forms and, with the exception of *WC1-11 *which is Type III, all other WC1 genomic and cDNA sequences in the various other clusters are classified as Type I. One cDNA clone included in those (CH533) contained archetypal WC1.1 sequence with a premature stop in the intracytoplasmic domain which should not affect tyrosine phosphorylation based on its location [49]; this sequence had been previously independently obtained as well [48]. For Type III, *WC1-11 *clustered with CH486 and their tail sequences were found to be most similar to swine WC1 supporting evidence described above for this unique type. To confirm this relatedness, swine WC1 sequences were included for comparison. There are five publicly available sequences for swine WC1 including one unique Domain 1 sequence and two unique intracytoplasmic tail sequences (swppWC1 and swWC1-29e1; GenBank accession numbers CAA67710 and CAA67709, respectively). Here we demonstrated bovine *WC1-11 *intracytoplasmic tail sequence is more closely related to swWC1-29e1 than to any other bovine WC1 intracytoplasmic tail region. However, the intracytoplasmic region of *WC1-11*, and its corresponding cDNA clone CH486, is strikingly longer than that of the other bovine WC1 genes and contains additional sequence (amino acids 790–837) that is not found even in swine WC1. Overall, the relationships established by comparing WC1 Domain 1 sequences (see Figure 7B) were largely maintained by the comparisons of tail sequences. However, some WC1 molecules, such as *WC1-4 *and *WC1-9*, have highly similar extracellular domains suggesting that they may bind the same ligand, but they differ in their tail structures, as illustrated in Figure 9B. This suggests that cells bearing *WC1-4 *or *WC1-9 *might differ in their functional responses (as occurs with other paired receptors [50]).

### Evidence for isoform generation

**Figure 10:**
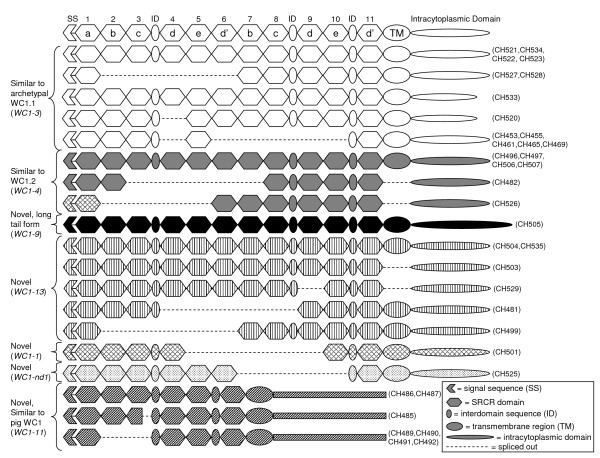
**WC1 isoform generation**. Schematic representations of 35 WC1 cDNA sequences derived from pooled mRNA from ex vivo and activated total PBMC taken from a single animal. The cDNA clone names upon which the schematics are based are indicated in parentheses to the right of the schematics. Where available, full-length cDNA sequences are shown with putative alternative splice variants shown below the full-length sequence of the same pattern. cDNA sequences were considered to be alternative splice variants of a particular full-length sequence when percent identity of the existing deduced amino acid sequence was ≥ 98%.

As mentioned above, RT-PCR products for WC1 transcripts yielded bands of approximately 2.7 kb and 2.2 kb in addition to the larger bands that most commonly contained full-length sequence. When the smaller bands were sequenced they were found to represent alternative splice variants. All 35 WC1 cDNA sequences obtained in this study are shown schematically with alternative splice variants aligned with non-spliced sequences to indicate the missing domains (Figure 10) providing evidence of isoform generation. Interestingly, on some occasions the larger bands were also found to contain transcripts representing alternative splice variants that lacked the transmembrane region and/or a single SRCR domain (e.g. CH503 and CH529). Spliced cDNA sequences were designated as a particular WC1 if the existing deduced amino acid sequence shared ≥ 98% identity with the corresponding full-length WC1 sequence.

There was no apparent pattern to WC1 splicing. It was most common for entire SRCR domains to be missing, though in one case (CH485) half of a domain was missing but it still appeared to result in a viable transcript. Most often five contiguous SRCR domains were missing so that the resulting transcript contained six SRCR domains; however, transcripts lacking anywhere from one to six full SRCR domains were identified. WC1 transcripts lacking the transmembrane regions (thus are putatively secreted molecules) were also identified as otherwise full-length WC1 sequences as well as for WC1 splice variants lacking either four or five SRCR domains. While two WC1 cDNA sequences (CH533 and CH520) have shorter intracytoplasmic domains due to premature stop codons, there was no evidence of alternative splicing within the intracytoplasmic domain.

## Discussion

To better characterize the bovine WC1 co-receptor family we annotated the WC1 genes in the bovine genome Btau_3.1 assembly and identified 13 WC1 genes distributed between two loci on chromosome 5. This included a novel WC1 gene that more closely resembles swine WC1 than it does previously identified ruminant WC1 genes. Work is ongoing to resolve the gaps in the genome assembly of chromosome 5. However, the number of WC1 genes in the multi-gene family agrees reasonably well with previous reports estimating the occurrence of many related WC1 genes based on Southern blots [11] and of 13 WC1 genes based on cDNA analysis of intracytoplasmic tail region transcripts [48] but is fewer than the 50 WC1 genes predicted for sheep by Southern blotting [46,47]. The distribution of WC1 genes between two loci is reminiscent of the two TCR gamma loci for cattle and sheep (TRG1 and TRG2) where it is predicted that a number of duplication events and a subsequent translocation event resulted in the formation of the TRG2 locus [51]. It is possible that similar events contributed to the formation of the WC1 loci because the distribution of WC1 genes based on sequence similarity does not support the idea that a single WC1 locus underwent duplication. Also, interestingly, the distribution of the three distinct exon-intron structures identified here for the WC1 genes (i.e. Type I, II or III) among the two loci also does not support locus duplication alone because the Type II and III genes are found only within a single locus.

We verified the gene annotations by amplifying and sequencing WC1 cDNA from bovine cells derived from a single animal. cDNA evidence confirmed the presence of at least a single functional gene, in an individual animal, for each of the three types of genes differentiated by their exon-intron structure. It is important to note that the profile of WC1 transcripts obtained from this individual animal is not necessarily representative of all WC1 genes expressed. This is despite using an experimental design in which cDNA from several conditions was pooled prior to PCR as well as using a variety of primers in an attempt to avoid biased amplification of particular WC1 transcripts. With regard to this, it was notable that transcripts representative of archetypal WC1.1, the only full-length sequence previously published [11], were not found to be abundant, to the extent that it was necessary to design a separate primer to preferentially amplify archetypal WC1.1 transcripts. Nevertheless, we confirmed the presence of 8 out of 13 WC1 genes based on cDNA sequences that corresponded to genomic sequences while cDNA evidence for *WC1-2*, *WC1-6*, *WC1-7*, *WC1-8 *and *WC1-12 *was not found. (Because many of those gene sequences are partial, it cannot be ruled out that cDNA evidence does exist for those genes but could not be classified as such at this point). Although all cDNA transcript sequences varied to some extent from corresponding genomic sequences, variations found between cDNA and related genomic sequences are most likely attributable to variation between animals due to single nucleotide polymorphisms. Indeed, even within a single animal there was preliminary evidence of allelic polymorphism (C.T.A. Herzig, unpublished data). Only two WC1 sequences derived from RT-PCR, CH525 and CH583, lacked any corresponding genomic sequence and were assigned the gene names of *WC1-nd1 *and *WC1-nd2*, respectively. The identification of a cDNA sequence lacking a corresponding genomic sequence could be a consequence of a gap in the genome sequence thus necessarily precluding the annotation of the corresponding gene. There is also evidence for copy number variation of WC1 genes among animals (G. Liu and J. Keele, personal communication, December 7, 2007) and this would also account for the observed differences.

Prior to these studies WC1 sequence corresponding to swine WC1 had not been identified in ruminants. The bovine swine-like *WC1-11 *reported here is structurally similar to WC1 in swine containing 6 SRCR domains, a transmembrane region and a long intracytoplasmic tail. It has been suggested that swine WC1 is the primitive version of its ruminant ortholog [29], so it could also be reasoned that in bovine *WC1-11 *is the most primitive of the ruminant WC1 genes. However, interestingly, the bovine *WC1-11 *has a very long intracytoplasmic region while swine WC1 genes have intracytoplasmic regions that are approximately the same length as those of the more classical bovine WC1 genes despite much dissimilarity in sequence [45]. Current evidence for the classical bovine WC1 intracytoplasmic tails indicates that both tyrosine and serine phosphorylation is important for activation signals and endocytosis, respectively [49]. It is possible that the presence of a tyrosine kinase phosphorylation motif within the unique portion of bovine *WC1-11 *intracytoplasmic region could result in a signaling and/or functional role that is distinct from other WC1 genes.

With regard to this, Wijngaard and co-workers identified and designated three distinct WC1 gene products as WC1.1, WC1.2 and WC1.3 based on reactivity with specific mAbs using WC1-transfected cells [45]. Based on those studies, WC1 bearing γδT cells were subsequently defined based on mAb reactivity as WC1.1^+^, WC1.2^+^, WC1.1^+^/WC1.2^+ ^or WC1.1^+^/WC1.3^+ ^wherein the WC1.3^+ ^population is only found as a subpopulation of WC1.1^+ ^cells. While the sequence for WC1.1 has been reported in its entirety, only limited putative amino acid sequence for Domains 1 and 2 and nucleotide sequence for segments of the intracytoplasmic tails for WC1.2 and WC1.3 have been reported. WC1.3 was unique with regard to its long intracytoplasmic tail [45] and that sequence can be found to correspond to Type II WC1 tail sequences here. However, based on our annotations no WC1 gene was identified that had the sequence reported by Wijngaard et al. [45] as that corresponding to Domain 1 of WC1.3; in fact similar sequence was instead found in Domain 6 of *WC1-4 *and *WC1-9*, and thus we suggest part of the published WC1.3 sequence is erroneous. Despite this problem it has already been shown that functionally distinct subpopulations of bovine γδT cells can be defined based on the presence of particular WC1 molecules that react with monoclonal antibodies recognizing WC1.1 or WC1.2 [2,14] and we now know that WC1 intracytoplasmic tails corresponding to the archetypal WC1.1 sequence play a critical role in signal transduction in response to antigen [49]. Thus, it is important to further evaluate the role of the long intracytoplasmic tail regions contained in Type II genes *WC1- 9, WC1-10, WC1-12 *and the swine-like Type III gene *WC1-11*. Because Domain 1 is the most diverse among WC1 SRCR domains, as shown here, it is possible that it serves as the pattern recognition portion of the WC1 molecule and could be a region where bacterial products are ligated as occurs for DMBT1 [52]. Therefore, pairings of particular WC1 Domain 1's with particular intracytoplasmic tail regions may be crucial to directing γδT cell responses and functions. Future studies will be targeted towards better understanding those relationships. For instance, transfection experiments with *WC1-4 *and *WC1-9 *would enable us to determine whether they bind the same ligands but their intracytoplasmic tails send different signals and thus result in differing functional responses.

Finally, the occurrence of a large variety of bovine WC1 molecules can be explained only in part by the number of WC1 genes since here we report evidence for extensive alternative splicing of bovine WC1 transcripts. In fact, all but one of the expressed WC1 genes we identified had corresponding splice variants. Immunoprecipitation of γδT cell membranes with anti-WC1 mAb results in a variety of bands including 144, 180, 200, 220, 240 and 300 kDa [10,12,53-57], lending support to the occurrence of multiple isoforms and/or swine-like *WC1-11 *on γδT cells. While the possibility remains that what appear to be alternative splice variants are instead genes that were not identified during the annotation process as a result of gaps in the genomic sequence, all but two alternative splice variants can be related to unspliced transcript sequences that are ≥ 98% identical. Moreover, previous reports indicate that swine and sheep WC1 orthologs [29,46] as well as other SRCR family immune system molecules (i.e. CD5, CD6 and CD163) produce transcripts that are alternatively spliced [22-27]. However, interestingly, unlike for CD6 and CD163 [23-26], WC1 intracytoplasmic tail length appears to be dictated by the particular gene encoding a transcript and not by alternative splicing.

It is notable that Domain 1, the putative ligand-binding portion, was never found to be missing as a result of alternative splicing. While this could be an artifact due to primer design, the forward primer was designed to anneal in the leader sequence and thus that explanation is unlikely. Precedence for multiple isoforms of a T cell co-receptor is shown by the two CD4-like genes in fish which differ from each other structurally [58,59]. The function of these smaller WC1 molecules with apparently intact Domain 1's and intracytoplasmic tails is unknown but intriguing. Because WC1 serves as a co-receptor on γδT cells, smaller WC1 molecules may be better able to co-cluster in the immune synapse with the shorter TCR chains (each being about 30 kDa). It is also possible that WC1 isoforms differ in their flexibility given that full-length WC1 molecules contain inter-domain or "hinge" regions following SRCR domains 3, 8, and 10, and this could affect interaction with the TCR. Differences in flexibility have been noted for functionally different immunoglobulin heavy chains with IgE and IgM lacking hinge regions. It is yet to be determined whether transcripts of the same gene but with different alternative splice variants are found expressed by an individual cell but perhaps WC1 splicing is initiated following interaction with its ligand.

## Conclusion

Based on annotations of the bovine genome we identified 13 members of the WC1 gene family and their organization within two loci. Many of those genes had not been previously described, including a gene coding for a putatively more primitive and smaller swine-like WC1 molecule. Furthermore, we provide cDNA evidence as verification for many of the annotated sequences as well as evidence for isoforms derived by alternative splicing and the suggestion of at least two more WC1 genes. It is possible that WC1 diversity contributes to functional differences that have been observed between γδT cell subpopulations and here we have demonstrated that diversity of WC1 molecules is attributable to a large, multi-gene family as well as to the fact that WC1 transcripts undergo extensive alternative splicing.

## Methods

### Genome annotation

In conjunction with the Bovine Genome Sequencing Consortium , manual annotation of the WC1 genes was performed using the Apollo Genome Annotation and Curation Tool, version 1.6.5 [60] and the bovine genome assembly Btau_3.1 [61]. Predicted gene models of putative WC1 genes were identified by performing a BLAST search of archetypal WC1.1 sequence against the Bovine Official Gene Set (called GLEAN). Predicted gene models were then analyzed using the Apollo software and the following actions were performed when necessary based on available EST or cDNA evidence: (i) models were checked for correct exon-intron structure, (ii) initiation and termination codons were identified, (iii) exons were either added or deleted if it was determined that the coding region in the predicted model was incorrect and (iv) predicted gene models were split when a single model encompassed more than one gene or merged when two models coded for a single gene. Predicted gene models identified from the BLAST search were considered pseudogenes when premature stop codons, frameshifts and/or a WC1 coding region exceeding approximately 60 kb occurred in areas where the sequence integrity was deemed adequate.

### Animals and cells

Blood was collected via jugular venipuncture into heparin from a single female Belted Galloway between the ages of 11 and 19 months. Animal use complied with federal guidelines and had IACUC approval. Peripheral blood mononuclear cells (PBMC) were isolated via density gradient centrifugation over Ficoll-Paque™ PLUS (GE Healthcare Bio-Sciences, Piscataway, NJ) according to the manufacturer's protocol. PBMC were cultured at 2.5 × 10^6 ^cells/ml with Concavalin A (ConA; 1.0 μg/ml; Sigma-Aldrich, St. Louis, MO) or leptospira antigen ([1], 0.5 μg/ml; sonicated whole cells of *L. borgpetersenii *serovar hardjo clone RZ33) in RPMI 1640 medium containing 10% heat-inactivated fetal bovine serum (HyClone, Logan, UT), 2 mM L-glutamine, 50 μM 2-mercaptoethanol and 50 μg/ml gentamicin at 37°C with 5% CO_2 _in air for six days.

### RNA isolation and RT-PCR

Pelleted ex vivo, ConA-activated or leptospira antigen-activated cells were resuspended in TRIzol (Invitrogen, Carlsbad, CA) and RNA was isolated according to the manufacturer's protocol. Reverse transcription (RT) was performed using 1 μg of total RNA, oligo dT primers and AMV reverse transcriptase (AMV RT kit; Promega, Madison, WI). 2 μl of pooled cDNA was used as template in subsequent polymerase chain reactions (PCR) using the Elongase Amplification system (Invitrogen) and a final concentration of 1.5 mM Mg^2+^. Generally, PCR was conducted using a forward primer (designated "WC1atg-for") designed to amplify all WC1 transcripts based on the annotations described here and an additional forward primer (designated "WC1full-forA") was used in separate reactions to preferentially amplify archetypal WC1.1 transcripts. Reverse primers (designated WC1groups1,2-rev, WC1group3-rev and WC1group4-rev) were designed to specifically amplify each of three sequence-related subgroups of WC1 transcripts based on the WC1 genes identified here. Primer sequences are in Table 2. Cycling parameters were 30s at 94°C, 30s at 52°C and 4 m 30s at 68°C for 35 cycles for all reactions. In a limited number of cases, WC1 Domain 1 only was amplified in PCR reactions using the WC1atg-for primer and a reverse primer which was designed within a conserved region to amplify Domain 1 of all known WC1 molecules, designated SRCR1-rev (Table 2). Cycling parameters for those reactions were 30s at 95°C, 1 m at 58°C and 1 m at 72°C for 30 cycles with an expected band size of approximately 530 bp. PCR products were analyzed on 1.2% TAE agarose gels, visualized using SYBR Safe (Invitrogen) or ethidium bromide and cloned into the pCR2.1 vector (Invitrogen) or the pCR-XL vector (Invitrogen) according to the manufacturer's protocol. A total of 35 full-length cDNA clones were sequenced commercially (GeneWiz, South Plainfield, NJ) in order to verify the insert identity and for subsequent sequence analysis.

### Sequence analyses

Sequencing of plasmids containing full-length WC1 cDNA was initially performed using T7 forward and M13 reverse primers. Subsequent sequencing reactions were performed using internal primers designed based on individual plasmid sequences (Table 2). Typically, four to six sequencing reactions were required to fully sequence a single WC1 transcript in the forward and reverse directions. Nucleotide sequences were aligned and consensus sequences were created using BioEdit version 7.0.5.3 [62]. WC1 sequences reported here that were derived from cloned RT-PCR products were submitted to GenBank ( see Table 5 for accession numbers), and classified based on annotated genomic sequences described herein. Other sequences used for comparisons in analyses included bovine archetypal WC1.1 (GenBank accession number X63723) and swine WC1 (GenBank accession numbers CAA67706 to CAA67710). Exon-intron structure schematics were based on alignments of cDNA and genomic DNA sequence using SIM4 [63] and visualized with LalnView .

Multiple sequence alignments were performed using ClustalW2 (; [64]) and the default parameters, refined by hand when necessary, and were visualized using JalView [65]. SRCR domain organization of alternatively spliced variants was determined using ClustalW2 analysis of deduced amino acid sequences to determine which domains were represented in each clone. Phylogenetic analyses were performed using deduced amino acid sequences of individual WC1 domains or tails, as indicated. Phylogenies were constructed in MEGA4 [66] using the neighbour-joining method [67] and the p-distance model for amino acid sequences and were tested using bootstrap analysis [68] with 1000 replications.

## Authors' contributions

CH carried out the annotations, molecular studies and the sequence analyses and helped to draft the manuscript. CB participated in the design of the study, interpretation of data, securing funding and helped to draft the manuscript.
